# Prevalence of Cardiovascular Risk Factors Among Patients With Diabetes Mellitus Type 2 at King Fahad University Hospital, Saudi Arabia

**DOI:** 10.7759/cureus.29489

**Published:** 2022-09-23

**Authors:** Noor-Ahmed Jatoi, Yasir A Elamin, Abir H Said, Basher Al-Namer, Fatimah A Al-Muallim, Fatimah F Al-Nemer, Fatimah M Al-Halal

**Affiliations:** 1 Internal Medicine (Vascular Medicine), King Fahad University Hospital, Imam Abdulrahman Bin Faisal University, Al-Khobar, SAU; 2 Internal Medicine (Diabetes and Endocrinology), King Fahad University Hospital, Imam Abdulrahman Bin Faisal University, Al-Khobar, SAU; 3 Internal Medicine (Pulmonolgy), King Fahad University Hospital, Imam AbdulRahman Bin Faisal University, Al-Khobar, SAU; 4 Internal Medicine, Imam AbdulRahman Bin Faisal University, Al-Khobar, SAU; 5 Internal Medicine, College of Medicine, Imam AbdulRahman Bin Faisal University, Al-Khobar, SAU

**Keywords:** haemodynamic measurements, dyslipedemia, hba1c, diabetes mellitus, cardiovascular risk factors

## Abstract

Background

Diabetes mellitus is considered a major risk factor for cardiovascular diseases. Patients with diabetes mellitus type 2 (DM-II) are at twice as high risk for the development of cardiovascular diseases than the general population. Thus, we aimed to assess the most prevalent cardiovascular risk (CVR) factors among DM-II patients in the Eastern province of Saudi Arabia.

Method

This is a cross-sectional, retrospective, and observational study conducted on DM-II patients at King Fahad University Hospital (KFUH) Al Khobar, Saudi Arabia, from January 2016 to December 2021. The total number of participants was 373 who were patients with DM-II. The patients' demographic information (age, sex, marital status, height, weight, body mass index (BMI), waist, hip circumference, and waist-hip ratio were calculated or obtained from hospital electronic records as were the CVR factors, age, gender, smoking habits, physical activity, BMI, haemodynamic measurements, glycosylated haemoglobin (HbA1C) levels and lipid profile.

The collected data were analyzed by using SPSS Statistics v.28 (IBM Corp., Armonk, NY). The descriptive statistics were reported using mean±SD for numerical data and relative frequencies (%) for categorical data. P < 0.05 were counted significant. Quantitative data were analyzed using the ANOVA test to compare the means of the three groups. Qualitative data were analyzed and compared using the chi-square test. Fisher’s exact test was also used to study the statistical significance of variables. Spearman rank correlation was used to study the relationship between HbA1C and other CV risk factors.

Results

The mean age was 58 (± 13) years; females were 57% of the sample. Around 92% were smokers, 84% had a sedentary lifestyle, 72% had dyslipidemia, 58% were obese, 30% were overweight, 58% reported poorly control of their diabetes, 50% had hypertension and 32% had pre-hypertension. Furthermore, 89% of participants had two or more CVR factors other than DM-II. We found a significant association between high body mass index, dyslipidemia, high systolic blood pressure and pulse pressure (p<0.05) with HbA1C.

Conclusion

The majority of participants had two or more cardiovascular risk factors in addition to DM-II. Poor control of DM-II and cardiovascular risk factors cannot be ignored and primary to tertiary prevention must be the top priority when managing the diabetic population in order to prevent devastating outcomes and progression of reversible morbidity.

## Introduction

People with diabetes mellitus comprise 10.5% of the world population of whom 90% have diabetes mellitus type-2 (DM-II), and the prevalence is expected to rise to reach 11.3% and 12.2% by 2030 and 2045 respectively [1]. Saudi Arabia is the second highest rate in the Middle East, and is ranked seventh globally. Alarmingly, this rate is continuously increasing [2].

DM-II is a chronic disease associated with devastating complications generated by endothelial alterations at different vascular levels. Hyperglycemia, hypertension, dyslipidaemia, obesity, physical inactivity, and smoking are the main risk factors that initiate the cascade of events leading to endothelial dysfunction, micro-vascular and macro-vascular complications specifically atherosclerotic cardiovascular (CV) diseases which include coronary artery disease, chronic peripheral artery disease, and cerebrovascular disease [3,4].

CV diseases are a great burden on patients with DM-II as these patients have twice as high a risk for the development of CV disease than people without diabetes. Furthermore, atherosclerotic CV disease is considered the primary cause of death in diabetic patients [5].

Studies have proven that long-term reduction of CV risk factors is associated with a lower risk of CV events and death [6,7]. Patients with multi-factorial risk factor control had survived for 7.9 years longer and had a lower rate of CV-related death by 62% than patients with usual care [6]. Hence, reducing the modifiable CV risk factors should be prioritized to reduce their related morbidity and mortality. Modifiable risk factors include uncontrolled diabetes, obesity, physical inactivity, poor glycemic control, smoking, high cholesterol and triglycerides [4].

Lowering the risk of CV disease requires that we have updated prevalence rates of CV risk factors among patients with DM-II. Thus, we aimed to assess the most and least prevalent CV risk factors among DM-II patients in the Eastern province of Saudi Arabia.

Purpose

The purpose of this study is to determine the prevalence of CV risk factors among DM-II patients at King Fahad university hospital Al-Khobar, Kingdom of Saudi Arabia.

## Materials and methods

Study design and population

This is a cross-sectional, retrospective and observational study conducted on patients with DM-II. The target population were all patients with DM-II, aged greater than 18 years who attended either diabetic, endocrine, or nephrology clinics at King Fahad University Hospital (KFUH) Al Khobar, Saudi Arabia, from January 2016 to December 2021. However, all patients with diabetes mellitus type 1 (DM-I), younger than 18 years, and pregnant women were excluded. The minimum acquired sample size is 177 patients (using the formula N=Z2*P(1-P)\d2) at a 95% confidence interval and a 5% margin of error. Using the previous criteria, a total of 373 patients were enrolled in the study. 

 Data collection

The patient demographic information (age, sex, marital status, height (cm), weight (kg), body mass index (weight (Kg)/Height (cm^2^)), waist circumference (cm) hip circumference (cm), waist-hip ratio, haemodynamic status, and glycaemic control status were obtained or calculated from hospital electronic records. Smoking status was categorized as current smoker, ex-smoker and non-smoker; physical activity status was categorized as active (≥75 minutes/week) of vigorous activity or ≥150 minutes/week of moderate activity, or non-active (<75 minutes/week of moderate activity) [8]. This study was conducted when the participants visited the clinic for a follow-up or through a phone call with the patients who hadn't an appointment during the study time period. 

The body mass index (BMI) was calculated using the following formula; BMI (kg/m^2^) = (weight in kilograms)/(height in metres)^2^. We divided the results into three groups, normal (18.5 - 24.9), overweight (25.0 - 29.9), obesity (≥30.0) [9]. Haemodynamic status includes systolic blood pressure (BP), diastolic (BP), pulse pressure (PP) and mean arterial pressure (MAP). Parameters were measured in the right arm with an automated digital sphygmomanometer (Omron, model HEM 705-CP, Omron Corporation, Shimogyo-Ku, Kyoto, Japan). BP measurements were grouped as either normal (≤ 120/80 mmHg), pre-hypertension (120/80 - 140/90 mmHg), and hypertension (≥ 140/90 mmHg) [10]. PP was calculated using the following equation (systolic BP - diastolic BP) which normally equals 40 mmHg [11] while MAP was calculated as diastolic BP+[1/3x (systolic BP - diastolic BP)] normally >60 mmHg [12]. Glycaemic control status was measured by fasting blood glucose (FBG) and glycosylated haemoglobin (HbA1C). Patients were divided according to their FBG to normal control ≤130 (mg/dl) or poor control >130 (mg/dl) [13]. Participants were classified into three groups depending on their HbA1c level. Group 1: patients with good control (<7%); Group 2: patients with fair control (between 7-8%); Group 3: patients with poor control (>8%) [14]. Lipid status was listed normal as the following values: total cholesterol (< 200 mg/dl), low-density lipoproteins, (LDL< 130 mg/dl), high-density lipoproteins (HDL >40 mg/dl), and triglycerides (TRG<150 mg/dl); and the abnormal values as the following: total cholesterol (≥ 200 mg/dl), LDL (≥ 130 mg/dl), HDL (≤ 40 mg/dl), and TRG (≥ 150 mg/dl) [15].

Statistical analysis

Data were analyzed by using SPSS Statistics for Windows, Version 28.0 (IBM SPSS Statistics for Windows, Version 28.0. Armonk, NY: IBM Corp). Descriptive data were reported using mean with standard deviation (SD) for numerical data and relative frequencies (percentages) for categorical data. The analyzed values were considered to be statistically significant if the P value was less than 0.05. Quantitative data were analyzed using the ANOVA test to compare the means of the three groups. Qualitative data were analyzed and compared using the chi-square test. Fisher’s exact test was also used to study the statistical significance of variables. Spearman rank correlation was used to study the relationship between HbA1C and other CV risk factors. 

Ethical consideration

Ethical clearance for the study was taken by the respective district medical officers in charge of data extraction from the QuadraMed system. For additional information, verbal consent was taken when there was contact with the patients.

## Results

Out of 373 participants, the mean age was 58 ± 13 (years), 57% were females, 73% were current smokers, 19% were ex-smokers, 84% had a sedentary lifestyle, 72% had dyslipidemia, 58% were obese, and 30% were overweight. Regarding glycemic control, 58% were poorly controlling their diabetes while 20% represented fair control according to HbA1C results. Furthermore, 50% had hypertension (HTN) whereas 32% had pre-HTN (Table 1).

**Table 1:** Clinical characteristics of the study population (n=373) Abbreviations: HTN, hypertension; HbA1C, hemoglobin A1c; LDL, low-density lipoprotein; HDL, high-density lipoprotein; TRG, triglycerides

Variables	Mean±SD (%)
Age (years)	58±13
Gender (M: F)	(43:57)
Height (cm)	162±9
Weight (kg)	83.7±18
Body mass index (kg/m2)	32.8±6.5
Body mass index status (normal: overweight: obese)	(12: 30: 58)
Waist (cm)	107±20
Hip (cm)	110±19
Waist hip ratio	0.99±0.18
Smoking status	
Never: current: ex-smoker	(8: 73: 19)
Physical activity	
Active: non-active	(16: 84)
Hemodynamic measurement	
Heart rate (bpm)	82±14
Systolic blood pressure (mmHg)	141±20
Diastolic blood pressure (mmHg)	82±12
Pulse pressure (mmHg)	61±17
Mean arterial pressure (mmHg)	104±16
HTN status (normal: pre HTN: HTN)	(18: 32: 50)
Laboratory	
Fast blood glucose (mg/dL)	171±66
Good: poor control	(31: 69)
HbA1C	9±5
Good: fair: poor control	(22: 20: 58)
Total cholesterol (mg/dL)	173±48
Total cholesterol (normal: abnormal)	(92: 8)
LDL (mg/dL)	105±42
LDL (normal: abnormal)	(56: 44)
HDL (mg/dL)	45± 15
HDL (normal: abnormal)	(72: 28)
TRG (mg/dL)	149± 89
TRG (normal: abnormal)	(64: 36)
Lipid status (normal: abnormal)	(28: 72)

Poor HbA1C was significantly associated with high BMI (p=0<001). The association of BMI with HbA1C revealed that a significantly high proportion of obese patients (65%) had poor glycemic control in comparison with normal and overweight patients (p=0.022). HbA1C level was also significantly associated with SBP and PP. It was found that those who had poor control had significantly high SPB (p=0.026) and PP (p=0.011) (Table 2).

**Table 2:** HbA1C categorized analysis Abbreviations: HTN, hypertension; HbA1C, haemoglobin A1C; LDL, low-density lipoprotein; HDL, high-density lipoprotein; TRG, triglycerides

Variable	HbA1C			P-value
	Good control mean±SD (%)	Fair control mean±SD (%)	Poor control mean±SD (%)	
Body mass index (kg/m^2)^	32±10	31±6	33±7	0.549
Body mass index status				0.022*
Normal	7	11	13	
Overweight	45	37	22	
Obese	48	52	65	
Waist (cm)	110±13	103±21	107±22	0.284
Hip (cm)	113±13	107±21	109±21	0.299
Waist-hip ratio	0.97±0.08	0.97±0.23	1.0±0.18	0.555
Smoking status				
Never	11	9	5	0.553
Current	74	71	78	
Ex-smoker	15	20	16	
Physical activity				
Active	22	10	17	0.221
Non-active	78	90	83	
Hemodynamic measurement				
Heart rate (bpm)	82±15	81±14	82±13	0.824
Systolic blood pressure (mmHg)	135±18	140±16	142±22	0.026*
Diastolic blood pressure (mmHg)	81±10	82±10	82±13	0.496
Pulse pressure (mmHg)	55±14	58±17	63±18	0.011*
Mean arterial pressure (mmHg)	99±14	104±13	105±17	0.099
HTN status				0.102
Normal	27	12	17	
Pre HTN	35	37	30	
HTN	38	51	53	
Dyslipidemia				
Total cholesterol (mg/dL)	167±46	167±52	177±47	0.166
LDL (mg/dL)	102±38	101±46	108±42	0.391
HDL (mg/dL)	43±14	48±15	45±15	0.17
TRG (mg/dL)	147±79	134±86	151±89	0.369

The correlation between BMI (p=0.022), SBP (p=0.043) and PP (p=0.049) with HbA1C were direct and statistically significant. There was a moderate and direct level of correlation between FBG and HbA1C (p=0<001). The correlation between total cholesterol and HbA1C was positive and statistically significant (p=0.006) (Table 3).

**Table 3:** Spearman rank correlations of variables against HbA1C Abbreviations: LDL, low-density lipoprotein; HDL, high-density lipoprotein; TRG, triglycerides

Variables	Correlation (r)	P-value
Age (years)	-0.059	0.279
Gender (M: F)	0.033	0.541
Height (cm)	-0.081	0.188
Weight (kg)	0.065	0.29
Body mass index (kg/m^2^)	0.144	0.022*
Waist (cm)	0.058	0.444
Hip (cm)	0.005	0.949
Waist-hip ratio	0.047	0.542
Smoking status		
Never: current: ex-smoker	0.008	0.886
Physical activity		
Active: non-active	-0.081	0.132
Hemodynamic measurement		
Heart rate (bpm)	0.033	0.585
Systolic blood pressure (mmHg)	0.11	0.043*
Diastolic blood pressure (mmHg)	0.054	0.321
Pulse pressure (mmHg)	0.124	0.049*
Mean arterial pressure (mmHg)	0.079	0.254
Glycemic control		
Fast blood glucose (mg/dL)	0.502	0.001*
Dyslipidemia		
Total cholesterol (mg/dL)	0.151	0.006*
LDL (mg/dL)	0.130	0.017*
HDL (mg/dL)	0.027	0.627
TRG (mg/dL)	0.092	0.095

Patients with no other CV risk factors besides DM-II represented only 4% of participants. The majority of participants had three other modifiable risk factors (30%). Overall, 89% had two or more other risk factors (Table 4).

**Table 4:** Distribution of other modifiable cardiovascular risk factors in DM-II patients

Cardiovascular risk factors	N (%)
No other risk factors	15 (4)
One other risk factor	28 (8)
Two other risk factors	73 (20)
Three other risk factors	111 (30)
Four other risk factors	91 (24)
Five other risk factors	55 (15)

## Discussion

The total number of patients with diabetes mellitus was 373 patients; the mean age was 58 (± 13) years, 57% were female, 92% had a smoking history, 84% had a sedentary lifestyle, 72% had dyslipidemia, 58% were obese, 58% had poor glycemic control while 20% had fair control and HTN represented 50% of patients. Most participants considered walking as an activity of their main daily exercise. In addition, more than half of DM-II patients had sedentary lifestyles explaining the high percentages of overweight and obesity. Dietary habits like consuming more carbohydrates, legumes, saturated fats, and salt can impact these percentages in the Saudi population particularly. Only 4% of patients had no CV risk factor other than DM-II. Likewise, 89% had two or more other CV risk factors. These findings are proving that the Saudi diabetic population are at high risk for developing CVD. 

HbA1C reveals the glycemic index of the haemoglobin for the past three months. Currently, the ADA recommends a goal of less than 7% to reduce CVD complications [16,5]. In our study, 58% of the participants had poor HbA1C control while 20% had fair HbA1C control. HbA1C level was significantly higher in participants with elevated SBP (mmHg), PP (mmHg), and high BMI which is similar to Hussein et al.'s (2020) findings with 77% of participants who had uncontrolled HbA1C [17]. Additionally, Peng et al. (2013) stated that patients with elevated HbA1C had a worse CV and metabolic risk than those with normal HbA1C [18]. Only 22% of the participants in our study has good HbA1C control, which was similar to a study done in the central region of Saudi Arabia by Kharal M et al. (2010), which showed only 19% with good HbA1C control [16]. Algamdi et al. (2021) found that with the increase in HbA1C concentration there was a higher risk for macrovascular, microvascular and mixed vascular events and a higher risk of death [19]. Therefore, it is urged to control HbA1C levels to be equal to or under 7%. 

Smoking increases cardiovascular morbidity and mortality and raises serum LDL. Fortunately, the risk is reduced gradually with smoking cessation [20]. The prevalence of poorly controlled HbA1C among smoker patients was high, although the association between smoking and poor HbA1C is not statistically significant. In poorly controlled HbA1C, 78% of patients were current smokers, while 16% of patients were ex-smokers, and only 5% of patients had never smoked. A similar finding was noted by Hussein et al. (2020) with 60% of patients with poorly controlled HbA1C were smokers [17]. Smoking was a dependent risk factor that might affect HbA1C only when other risk factors were present.

Dyslipidemia is a condition of abnormal metabolic increase of lipoprotein causing a constant increase in the plasmatic concentration of cholesterol along with triglyceride [21]. Our study shows a statistically significant positive correlation (p<0.05) between poor control of HbA1C and abnormal total cholesterol and abnormal LDL with a prevalence of 8% and 44% respectively. This matches the result of Artha et al. (2019), which found a significant positive correlation between HbA1C level and lipid profile [15]. On the contrary, Hussein et al. (2020) result showed no significant relationship between HbA1C and lipid profile [17]. In our study, 92% had normal total cholesterol, equivalent to a study made in Riyadh by Al Slail et al. (2015), which presented that optimal total cholesterol was present in 82% of participants [21]. Ideally, TRG should be significantly high but that did not appear in our study, the reason might be because the participants were using lipid-lowering drugs.

Physical activity has been reported to have an impact on decreasing FBG and HbA1C levels [22]. Inactivity had a positive association with the progress of CVD in the DM-II population [23]. In our study, the prevalence of poorly controlled HbA1C among inactive participants was higher but, physical activity was not statistically significant. Currently, there is no study mentioning the relationship between sedentary lifestyles and increased HbA1C.

There is a significant association between high BMI and poor control of HbA1C. 65% of the patients who had poor HbA1C were obese. These findings could be explained by visceral fat accumulation that eventually increases insulin resistance, resulting in diabetes [17]. This positive association matches the findings of Hussein et al. (2020) with a large prospective cohort study which was conducted on 17,638 and found that 80% of participants with obesity had elevated HbA1C [17].

We found a significant association between SBP, PP and HbA1C. The positive association between high BP and high HbA1C was also noted by several other studies as in Hussein e al. (2020) and Alavudeen et al. (2013) [17, 24]. This link might be attributed to the presence of shared risk factors and inflammatory processes seen in both HTN and hyperglycemia [17,25]. In our study, 50% of diabetic patients had uncontrolled BP. This was significantly higher than in the southern region of Saudi Arabia where Alavudeen et al. (2013) stated that HTN among only 24.71% of patients with DM-II [24]. On the other hand, global studies had a higher prevalence estimated at 60.25%, 74.5% and 77.6% in Egypt, north Catalonia and Sri Lanka respectively [17,26,27].

Male sex and age are both considered non-modifiable CVD risk factors [28]. Both of these risk factors were not statistically significant in our study. However, we found that more than half of our population were females (57%) with a mean age of 58 years [23,29,30]. Afroz et al. (2019) stated being a female in addition to other factors was related to poor glycemic control and found that the middle-aged population had poor glycemic control [14]. In the past three decades, there has been a decrease in both total and CVD mortality in diabetic male patients. On the other hand, diabetic females’ mortality showed no decline [30]. However, the mortality rate of male patients with diabetes is still higher. This difference in mortality could be given to biological, physiological and behavioural factors. Moreover, this development could be caused by the way physicians have been approaching female patient management as it has been shown that women are less aggressively managed for many CHD factors. Consequently, a significantly higher proportion of women did not reach recommended target levels of HbA1c [30].

Strengths and limitations

To the best of our knowledge, no studies were conducted in the Eastern province of Saudi Arabia regarding the assessment of cardiovascular risk factors among DM-II patients. Some cardiovascular risk factors such as socioeconomic status, alcohol consumption, and adherence to medication regimens were not considered in our study.

Recommendations

Prevalence of CV risk factors will be more representative of the population, if this study, focusing on the Eastern region of Saudi Arabia, could be included in the meta-analysis that should be done if more similar studies were published emphasizing the prevalence of CV risk factors among DM-II patients in the west and north regions of Saudi Arabia. Furthermore, the prevalence of a sedentary lifestyle among diabetics should be under consideration in future studies.

## Conclusions

One-third of participants had three other CV risk factors in addition to DM-II. The most prevalent CV risk factors were smoking, sedentary lifestyle, dyslipidemia, obesity and HTN. High BMI, systolic BP and PP all had a significant association with poor control of diabetes. The Eastern region of KSA had a higher prevalence of HTN than the southern region. The association between poor control of DM-II and modifiable CV risk factors can not be ignored and primary to tertiary prevention must be a top priority when managing DM-II patients in order to prevent devastating outcomes and progression of morbidity which can be reversed. There is a significant association between high BMI, high systolic BP and PP with elevated HbA1C.
